# Nanotherapy Targeting miR-10b Improves Survival in Orthotopic Glioblastoma Models

**DOI:** 10.3390/jfb17010015

**Published:** 2025-12-26

**Authors:** Bryan D. Kim, Ming Chen, Sujan K. Mondal, Elizabeth Kenyon, Christiane L. Mallett, Ana deCarvalho, Zdravka Medarova, Anna Moore

**Affiliations:** 1 Precision Health Program, Michigan State University, East Lansing, MI 48824, USA; 2 Department of Biomedical Engineering, College of Engineering, Michigan State University, East Lansing, MI 48824, USA; 3 Department of Radiology, College of Human Medicine, Michigan State University, East Lansing, MI 48824, USA; 4 Institute for Quantitative Health Science and Engineering, Michigan State University, East Lansing, MI 48824, USA; 5 Henry Ford Health, Detroit, MI 48202, USA; 6 TransCode Therapeutics Inc., Woburn, MA 02458, USA

**Keywords:** glioblastoma, miRNA, nanoparticle, image-guided therapy

## Abstract

Glioblastoma (GBM) is the most aggressive primary cancer with poor survival. In the absence of an effective treatment and a high probability of recurrence, new therapeutic approaches are urgently needed. This study focused on targeting microRNA-10b (miR-10b) highly expressed in GBM cells that has been identified as one of the key drivers of GBM progression. Inhibiting miR-10b using antisense oligonucleotides (ASOs) has shown promise, but its delivery is challenging due to short circulation half-life, degradation by nucleases, and limited blood–brain barrier (BBB) permeability. To overcome these barriers, we employed a magnetic nanoparticle (MN) platform to deliver anti-miR-10b ASOs (MN-anti-miR10b). In addition to serving as a delivery vehicle, these nanoparticles can be used for monitoring delivery using magnetic resonance imaging (MRI). In therapeutic studies in orthotopic models of GBM presented here we used MN-anti-miR10b as well as TTX-MC138, a clinically tested anti-miR10b nanotherapeutic now in Phase I trials in patients with solid (non-GBM) cancers. Both formulations showed efficient delivery, as demonstrated by imaging and improved survival, leading to target inhibition and increased apoptosis. This approach may offer a novel strategy for delivering therapeutics to GBM and improving patient outcomes in one of the most aggressive and treatment-resistant forms of brain cancer.

## 1. Introduction

Glioblastoma (GBM) is the most aggressive and common primary cancer of the central nervous system (CNS) with poor survival rates. Recent statistics indicate an estimated mean survival of 15 months from diagnosis, with an overall five-year survival rate of approximately 5%, despite the standard of care [1]. GBMs are commonly resected surgically to the maximum extent feasible, followed by radiation therapy (RT) and chemotherapy with DNA-alkylating agents, such as temozolomide (TMZ), the only FDA-approved modality that constitutes first-line therapy for the treatment of GBM [2]. In addition to the precarious location of GBM tumors, the blood–brain barrier (BBB) while disrupted in tumors still often limits the delivery of therapeutics to tumors [3].

Aberrant expression of microRNAs (miRNAs), a class of short non-coding RNAs that post-transcriptionally modulate protein expression, has recently been implicated in the tumorigenesis of various cancers, including GBM [4]. Among these, microRNA-10b (miR-10b) has been identified as a key driver of GBM progression. It is highly expressed in tumor cells but is absent in normal neuroglial cells of the brain [5,6,7,8]. In GBM, miR-10b plays a direct role in promoting cell invasion and migration, supporting tumor growth, and contributing to resistance to apoptosis [5,7,8,9,10,11,12,13]. Most importantly, miR-10b is essential for GBM viability, and its inhibition by antisense oligonucleotides is detrimental to the tumor, but has no obvious negative effects on normal neural cells [5,14]. Previous studies have shown that the inhibition of miR-10b restores the expression of pro-apoptotic genes and decreases the growth of GBM cells through apoptosis and/or cell cycle arrest [6]. This inhibition can be achieved by delivering antisense oligonucleotides (ASO) to tumors. However, delivery of an active ASO molecule to the cell of interest represents a significant challenge because of its inability to cross the cell membrane due to charge-charge repulsion [15], short half-life and digestion by nucleases [16,17], entrapment in endosomes [18], off-target effects, and activation of immune responses [19]. Previous studies investigating various delivery strategies, including lipid particles, viral vectors, and polymers, have largely failed to effectively target tumors, particularly GBM, owing to limitations related to large particle size (e.g., as noted in [20]), poor biodistribution to the brain, induction of immune responses, and inability to cross the blood–brain barrier (BBB) [21]. Importantly, most published studies with these carriers have been conducted in vitro, while in vivo experiments have been performed using subcutaneous GBM tumor models, which lack clinical relevance [22,23,24,25]. A recent work in orthotopic tumors using CRISPR/Cas9 system to edit miR-10b showed potential in animal models after intracerebroventricular (ICV) injections [26]. However, clinical implementation of this approach would require a neurosurgical implant, which carries risks of infection, hemorrhage, and mechanical complications. In addition, ICV delivery is limited for areas near the ventricles making it unsuitable for tumor tissues located deep within the brain. Large lipid nanoparticles encapsulating the editing system used in this study are too large to efficiently diffuse through the tissue. Finally, a recent study showed that CRISPR/Cas9 can cause large DNA rearrangements potentially triggering cancer and that CRISPR-induced double-strand DNA breaks not only create intended edits but can also facilitate retroelement insertions at both the target site and elsewhere in the genome [27]. Another major shortcoming of existing delivery systems is the lack of imaging capability, which is essential for verifying whether the therapeutic agent reaches the tumor and for monitoring treatment response. This limitation underscores the urgent need for a next-generation multifunctional delivery platform that is capable of efficiently crossing the BBB, delivering ASOs to GBM cells, and simultaneously providing real-time imaging to monitor biodistribution and therapeutic efficacy.

To overcome the shortcomings of prior research and develop a reliable delivery system, we employed a magnetic nanoparticle (MN) platform to deliver anti-miR-10b ASOs (locked nucleic acids, LNA-based antagomirs) to tumors (termed MN-anti-miR10b [28]). These nanoparticles consist of an iron oxide core, enabling magnetic resonance imaging (MRI) for their delivery to the target site. The nanoparticles are coated with dextran, to which the ASO oligonucleotides are conjugated via a labile disulfide linker that ensures its release in the low-pH microenvironment of the tumor cell cytoplasm and binding to its target, miR-10b. We have previously used the MN platform for delivery of a 15-mer anti-miR10b ASO to both primary tumors and metastases in other miR-10b-expressing cancers, such as breast cancer, leading to arrest, and in some cases, elimination of metastatic growth in small and large animals (companion cats) [29,30,31,32]. Building on the success of these studies, this MN-anti-miR10b formulation became the foundation for an investigational therapeutic (TTX-MC138) currently under evaluation in a Phase I clinical trial in patients with advanced solid (non-GBM) cancers (NCT06260774).

In the present study, we investigated the therapeutic effect of MN-anti-miR10b containing 23-mer ASO, as well as the clinically tested TTX-MC138, in GBM animal models. First, in addition to demonstrating the profound effect of MN-anti-miR10b on viability of conventional immortalized human and murine cell lines that we reported earlier [28] in this study we showed that this loss of viability resulted in significant downregulation of the target (miR-10b) not only in these conventional cell lines but also in patient-derived cell lines with various degree of miR-10b expression, O^6^-methylguanine DNA-methyltransferase (MGMT) promoter status or response to TMZ or radiation therapy. Next, we confirmed MN-anti-miR10b and TTX-MC138 delivery to orthotopic GBM tumors after systemic administration, followed by therapeutic studies in vivo, where we demonstrated improved survival accompanied by inhibition of the target and induction of apoptosis. We believe that the proposed MN platform holds significant translational potential for GBM patients, a population for whom therapeutic options remain extremely limited. By addressing critical barriers to effective treatment—such as drug delivery across the blood–brain barrier and biomarker-specific targeting—this approach may offer a novel and impactful strategy for delivering therapeutics to GBM and improving patient outcomes in one of the most aggressive and treatment-resistant forms of brain cancer.

## 2. Materials and Methods

### 2.1. Cell Culture

Luciferase-expressing human glioblastoma, U251 (RRID:CVCL_0021) and LN229 (RRID:CVCL_0393), and murine glioblastoma GL261 cell lines were cultured in Dulbecco’s Modified Eagle Medium (DMEM) (Gibco, Jenks, OK, USA) containing 4.5 g/L D-Glucose, L-Glutamine, and 110 mg/L Sodium Pyruvate. DMEM was supplemented with 10% Fetal Bovine Serum (FBS) (Qiagen, Germantown, MD, USA) and 1% penicillin/streptomycin (Gibco Jenks, OK, USA). The cells were passaged by trypsinization with 0.25% trypsin/ethylenediaminetetraacetic acid (EDTA; Gibco, Jenks, OK, USA). All the cells were maintained at 37 °C with 5% CO_2_.

Human patient-derived luciferase-expressing GBM cancer stem cells (GSCs) were maintained in Dulbecco’s Modified Eagle’s Medium F-12 (DMEM/F-12) (Gibco) at 37 °C and 5% CO_2_ as 3D neurospheres [33]. DMEM/F12 was supplemented with N2 Supplement (Gibco, Jenks, OK, USA), bovine serum albumin (BSA) (Sigma-Aldrich, Saint Louis, MO, USA), gentamicin (Gibco), and antibiotic/antimycotic (Gibco, Jenks, OK, USA). Neurospheres were induced in supplemented DMEM/F12 with the addition of EGF (Peprotech, Cranbury, NJ, USA) and FGF (Peprotech, Cranbury, NJ, USA). A list of cells reflecting their properties is provided in Supplementary Table S1. U251 cells and patient-derived GSCs expressing firefly luciferase were obtained from Dr. Ana deCarvalho (Henry Ford Health, Detroit, MI, USA).

### 2.2. Therapeutic Synthesis

MN-anti-miR10b therapeutic was synthesized as previously described [28]. The therapeutic consists of a magnetic iron oxide nanoparticle core (MN) with a crosslinked dextran coating, which is functionalized by amine moieties. The aminated dextran coating was conjugated to a Cy5.5 optical dye, enabling near-infrared optical imaging, and an anti-miR-10b locked nucleic acid (LNA) 23-mer oligonucleotide. Briefly, the anti-miR-10b antagomir, directed against miRNA-10b, and a mismatch scrambled (Scr) sequence, with the 5′-Thiol-Modifier C6 disulfide (5′-ThioMC6) inserted into both sequences for conjugation to magnetic nanoparticles, were synthesized by Integrated DNA Technologies (Coralville, IA, USA). The thiol-modified oligonucleotides were activated by treatment with 3% TCEP (Tris(2-carboxyethyl)phosphine hydrochloride (Thermo Fisher Scientific, Rockford, IL, USA)), followed by purification with ammonium acetate/ethanol precipitation before conjugation to the nanoparticles, as described previously [28]. Oligonucleotide loading efficiency was the same for both experimental (MN-anti-miR10b) and control (MN-Scr) therapeutics that were used for in vitro and in vivo studies. In addition, we used TTX-MC138, an oligo-nanoparticle conjugate devoid of Cy5.5 optical dye provided by TransCode Therapeutics Inc. (Woburn, MA, USA) with the same oligonucleotide loading efficiency. All preparations were characterized and described in our previous publications [28,29]

### 2.3. Analysis of Plasma Stability

The stability of the therapeutic was investigated in rodent (rat) plasma. TTX-MC138 (40 µg/mL) was added to plasma and incubated for 30 min, 2 h, 24 h, 72 h and 96 h. Plasma was then mixed with the Novex™ TBE-Urea Sample Buffer (2X) (Cat#LC6876; Thermo Fisher Scientific, Waltham, MA, USA), heated at 70 °C for 3 min and applied onto 15% Mini-PROTEAN^®^ TBE-Urea Gel (Cat#4566053; Bio-Rad Laboratories, Ann Arbor, MI, USA). Oligonucleotides were detected by staining with the 0.02% methylene blue staining solution (Cat# 1808-50 mL; Millipore Sigma, Burlington, MA, USA). Free antisense oligonucleotides were used as a standard.

### 2.4. Pharmacokinetics Studies

Pharmacokinetics studies were performed by the Charles River Laboratories (CRL) Edinburgh Ltd. (East Lothian, UK) and Charles River Laboratories Montreal ULC (Sherbrooke, QC, Canada). Rats (Sprague Dawley, n = 3/data point, Charles River Laboratories Sherbrooke, QC, Canada) were intravenously injected with TTX-MC138 (30 mg oligo/kg) and sacrificed 5 min, 15 min, 30 min, 1 h, 2 h, 4 h, 6 h, 24 h, 48 h and 96 h after injection. Plasma samples were analyzed for the presence of iron and anti-miR10b antisense oligonucleotide. Analytical procedure for the analysis of iron in rat plasma was developed by CRL prior to the analysis. In the analytical procedure, samples of rat plasma were appropriately diluted and analyzed by Inductively Coupled Plasma Mass Spectrometry (ICP-MS) using Perkin Elmer NexION 350X ICP-MS Analyzer (PerkinElmer, Hopkinton, MA, USA). Analytical procedure for the analysis of anti-miR10b antisense oligonucleotide in rat plasma was developed by CRL prior to the analysis. Sample preparation involved the affinity purification extraction of TTX-MC138 from rat plasma. The analyte was identified and quantified using reversed-phase Ultra-High Performance Liquid Chromatography (UHPLC) with MS/MS detection (Agilent Technologies, Santa Clara, CA, USA). Concentrations of analyte were determined using the peak area ratio response of the analyte/internal standard (anti-miR-10b antisense oligo) with calibration curve linearity established using a weighted 1/x^2^ linear (y = mx + b) least squares regression analysis.

### 2.5. Cell Treatment and Real-Time Quantitative RT-qPCR

All cell lines mentioned in Section 2.1 were treated with IC50 concentrations of MN-anti-miR10b (established in), MN-Scr (corresponding concentration of the Scr oligo) or PBS for 48hrs, washed and subjected to RT-qPCR. The total RNA from the cells was purified using an miRNeasy Mini Kit (Qiagen, Germantown, MD, USA) and quantified by spectrophotometry (Nanodrop). cDNA was synthesized using miScript II RT Kit (Qiagen, Germantown, MD, USA). Target miRNA was amplified and measured using Bio-Rad CFX96 Real Time System. The protocol was performed for 40 cycles, comprising 95 °C for 15 min, 94 °C for 15 s, 57 °C for 30 s. miRNA expression was determined using TB Green Kit (Takara Bio USA, San Jose, CA, USA). U6 expression levels were used as the quantitative internal control. For precise quantification, the miRNA expression level of each sample was normalized using the expression of the U6 for miRNAs. Data were shown as fold change (2^−ΔΔCt^).

### 2.6. In Vitro Apoptosis Assays

To assay apoptosis in vitro, the cells were stained with terminal deoxynucleotidyl transferase (TdT)-mediated dUTP nick end-labeling (TUNEL) and counterstained with 4′,6-diamidino-2-phenylindole (DAPI). TUNEL staining was performed according to the manufacturer’s protocol using the DeadEnd Fluorometric TUNEL System (Promega, Madison, WI, USA). The cells were also incubated with DAPI (Molecular Probes, Eugene, OR, USA) for 10 min at room temperature and then rinsed with distilled water. Glass cover slips were mounted on glass slides using mounting medium. The TUNEL and DAPI staining patterns were acquired using a fluorescence microscope (Nikon Eclipse 50i, Tokyo, Japan, RRID:SCR_027281) and analyzed using ImageJ2 software (NIH, Bethesda, MD, USA).

Alternatively, cells were stained with Annexin V conjugated with fluorescein isothiocyanate and Propidium Iodide (PI) (Apoptosis Detection Kit, Biolegend, San Diego, CA, USA) according to manufacturer’s instructions for 15 min in the dark at room temperature and analyzed using an Accuri C6 flow cytometer (BD Biosciences, San Jose, CA, USA). Data were quantified by Flowjo 10 (BD Biosciences, San Jose, CA, USA) software.

### 2.7. Orthotopic Tumor Implantation

Nude athymic mice (RRID:IMSR_JAX:007850) were orthotopically injected with 500,000 cells in 5 µL of PBS using a free-hand method as previously described [34]. Briefly, U251 cells were grown in culture and prepared at the appropriate concentration for injection into a PBS solution after dissociation with trypsin. HF3016 neurospheres were dissociated into single cells in PBS using gentle pipetting. Single cells were then pelleted and prepared at an appropriate concentration in PBS for intracranial injection. Mice were anesthetized by intraperitoneal administration of ketamine and xylazine (100 and 10 mg/kg, respectively). Extended-release buprenorphine (3.25 mg/kg) was subcutaneously injected as an analgesic. After disinfection of the surgical site with betadine and 70% isopropyl alcohol, an incision was made along the midline of the head, and the periosteum was removed. The skull was dried using a solution of 3% hydrogen peroxide to enhance visualization of the bregma. The drilling site was measured −2.5 mm medial/lateral and −1 mm anterior/posterior from bregma, and a hole was drilled using a micro surgical drill. The Hamilton syringe fitted with a stopper was set to a needle depth of 3 mm. During injection, the needle set to 3 mm was completely inserted into the drill site and then retracted approximately 0.5 mm to provide an area for the injected cells to accumulate. Cells were injected into the drill site over a duration of a minute. After the injection, the needle was held at the injection depth for one minute before slowly retracting it to minimize leakage of the injected cells. The drill site was then closed using bone wax and the incision was closed using a vet bond. Topical lidocaine was administered to the incision site as an additional analgesic. The mice were monitored until they fully recovered and were ambulatory. Tumor growth was monitored using in vivo bioluminescence imaging (BLI), as described below.

### 2.8. In Vivo Imaging of Tumor-Bearing Animals

In vivo bioluminescence and fluorescence imaging of tumor-bearing mice were performed to monitor tumor growth and nanoparticle accumulation, respectively, using an In Vivo Imaging System (IVIS Spectrum, Revvity, Waltham, MA, USA, RRID:SCR_018621). For the analysis of therapeutic accumulation in cancer lesions, ROIs were drawn around each tumor using LivingImage 4.5 software (RRID:SCR_014247). Mice were sacrificed following the final round of imaging, and tumors and other major organs were extracted and imaged ex vivo using an IVIS Spectrum imaging system.

All MR images were acquired with a 7 Tesla Biospec 70/30 USR (Bruker, Billerica, MA, USA, RRID:SCR_027282). Animals were imaged before and 24 h after intravenous injection of the therapeutic agent using the following parameters: 3D T2* weighted FLASH sequence with TR = 30 ms, TE = 10 ms, FOV 20 × 15 × 12 mm, resolution 100 µm isotropic, 50 min scan. We also acquired a T2* map, with TR = 800 ms, TE = 3.5 ms, FOV 20 × 20 mm, 5 coronal slices, no slice gap, resolution 100 µm in plane, and 4 min scan. Ten positive echo images were acquired with a 5 ms echo time spacing. Image analysis was conducted using Paravision 360 v3.1 (Bruker, Billerica, MA, USA, RRID:SCR_025295).

### 2.9. In Vivo Therapeutic Studies in Orthotopic GBM Models

Nude athymic mice were implanted with the human U251 glioblastoma model as described above. Treatment started on day 7 post tumor implantation after randomization into treatment groups, which was based on the baseline BLI measurements, typically 10^5^–10^6^ photons/s/cm^2^/sr (Supplementary Figure S1). Two studies were performed using the following formulations and regimens: (1) MN-anti-miR10b (10 mg oligo/kg, n = 6), MN-Scr (10 mg oligo/kg, n = 5), or PBS (n = 6) injected intravenously; (2) TTX-MC138 (10 mg oligo/kg, n = 6), TTX (20 mg Fe/kg, n = 7), and PBS (n = 7) injected intravenously. The amount of iron in TTX control group was adjusted to the amount of iron in experimental groups. The injection dose was selected based on our previous studies in tumor bearing animals [30,31,32,35]. During treatment, the mice weight was taken at each time point, and animals were monitored for tumor growth using BLI. Accumulation of MN-anti-miR10b and MN-Scr in tumors was monitored by in vivo fluorescence imaging (FLI) using the IVIS Spectrum. Treatment was continued for 8–10 weeks or until the animals became moribund.

For histopathology, major organs (liver, kidney, heart, spleen and lungs) were excised, embedded in Tissue-Tek OCT compound (Sakura Finetek), snap-frozen in liquid nitrogen and cut into 7 µm sections. The tissues were stained with hematoxylin and eosin (H&E, Thermo Fisher Scientific, Waltham, MA, USA). Images were acquired using an Aperio Versa 8 Brightfield & Fluorescence imaging system (Leica Biosystems, Buffalo Grove, IL, USA).

Animal studies were approved by the Institutional Animal Care and Use Committee (IACUC) of Michigan State University (protocol # PROTO202300364) and were in compliance with the National Institutes of Health Guide for the Care and Use of Laboratory Animals.

### 2.10. RNA Isolation and Gene Expression Analysis

Total RNA, including miRNAs, was isolated from brain sections (10 × 10 µm sections) containing tumors using the miRNeasy Mini Kit (Qiagen, Germantown, MD, USA) according to the manufacturer’s protocol. Total RNA (500 ng) was converted to cDNA using a Mir-X™ miRNA qRT-PCR TB Green Kit (Takara Bio USA, San Jose, CA, USA). cDNA was diluted 1:50 for miR-10b qPCR analysis relative to the human TATA Binding protein. Data are shown as fold change (2^−ΔΔCt^).

### 2.11. Laser Ablation Inductively Coupled Plasma Mass Spectrometry

Fresh frozen brains embedded in OCT were cryosectioned (10 µm) and placed on glass slides for elemental analysis using laser ablation inductively coupled plasma time-of-flight mass spectrometry (LA-ICP-TOF-MS) on a Bio Image 266 (Elemental Scientific Lasers, Bozeman, MT, USA). Tissue sections and gelatin containing IV-74434 or Na_2_HPO_4_ were ablated with a spot size of 10 or 20 µm using 60% laser power and a 125 Hz repetition rate. Iron and phosphorus concentrations within the tissues were determined relative to the respective gelatin standards.

### 2.12. Ex Vivo Fluorescence and Light Microscopy

To visualize MN-anti-miR10b in tissues, fresh frozen brains embedded in OCT were cryosectioned (10 µm), placed on glass slides, and fixed in 4% paraformaldehyde (PFA) for 15 min. The slides were then rinsed with PBS and mounted with Fluoromount-G mounting medium (Southern Biotech, Birmingham, AL, USA) containing 4′,6-diamidino-2-phenylindole (DAPI). Slides were then imaged in Cy5.5 and DAPI channels using a Nikon Eclipse 50i microscope.

To assess the accumulation of TTX-MC138 which did not contain Cy5.5, Prussian Blue Staining was used. Briefly, 10 µm tumor sections fixed with 4% PFA (Thermo Fisher Scientific, Rockford, IL, USA for 15 min were incubated with Prussian Blue solution containing equal volumes of potassium ferrocyanide (Abcam, Waltham, MA, USA) and 2% hydrochloric acid (Abcam) for 20 min and counterstained with nuclear fast red (Abcam). Slides were then imaged under bright-field illumination using a Nikon Eclipse 50i microscope (Nikon, Melville, NY, USA).

### 2.13. Ex Vivo TUNEL Assay

TUNEL staining was performed using a fluorescent (594 nm) TUNEL Assay Kit (Cell Signaling Technology, Danvers, MA, USA). Fresh frozen tumor-bearing brains were sectioned into 10 µm sections and fixed with 4% PFA. Tissues were counterstained and coverslipped using DAPI Fluoromount-G mounting medium. Slides were imaged with a Zeiss Axioscan 7 (Oberkochen, Germany, RRID:SCR_027284) in TUNEL (excitation 594 nm, emission 614 nm), DAPI, and Cy5.5 channels. TUNEL staining of the tumor region was performed using QuPath (RRID:SCR_018257) [36]. Briefly, an ROI was drawn around the tumor region, and individual cells were detected using the Cell Detection function in QuPath based on the DAPI channel. Cells were then categorized as TUNEL staining positive or-negative based on the intensity of TUNEL staining in the nuclear region of the cells.

### 2.14. Statistical Analysis

Statistical analysis was performed using the GraphPad Prism software (version 10, GraphPad Software Inc., San Diego, CA, USA, RRID:SCR_002798). Data are presented as mean ± SEM. Statistical comparisons were performed using Student’s *t*-test. In all cases, a value of *p* < 0.05 was considered significant. Survival was determined using Kaplan–Meier survival analysis and the log-rank (Mantel–Cox) test.

## 3. Results

### 3.1. Stability and Pharmacokinetics

**Figure 1:**
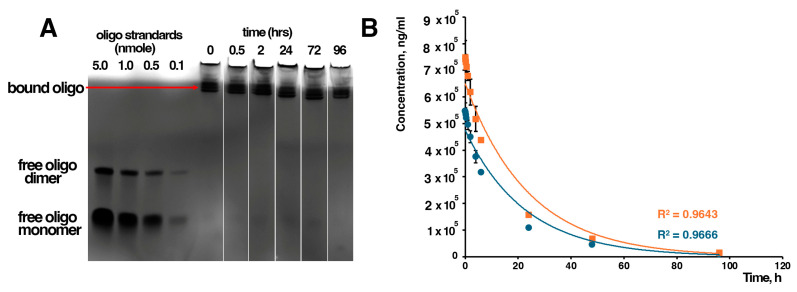
(**A**) Stability of TTX-MC138 in rat plasma was tested up to 96 h demonstrating no evidence of free oligo at any time point. (**B**) Pharmacokinetics in rats up to 96 h demonstrated two-phase decay indicating a strong positive correlation between oligo (blue line) and iron concentration (orange line)–time profiles (Pearson’s r = 0.99999, m = 10, *p* < 0.0001) consistent with the oligo remaining associated with the iron oxide nanoparticle.

The stability of the therapeutic was evaluated in rat plasma following incubation for up to 96 h. As shown in Figure 1A, no evidence of free oligonucleotides (either monomer or dimer) was detected at any time point, indicating that the therapeutic remains a stable complex between the iron oxide nanoparticles (TTX) and the oligonucleotide in plasma. These results were further confirmed by pharmacokinetic studies (Figure 1B and Table 1). Based on the pharmacokinetic parameters presented in Table 1, the oligo–iron oxide nanoparticle conjugate exhibited clear biphasic (two-phase) pharmacokinetics, consistent with a distribution phase followed by a slower elimination phase. Both the oligo and iron components show very similar distribution rate constants (kα ≈ 0.145 h^−1^) and corresponding distribution half-lives of ~4.8 h, indicating rapid and coordinated early tissue distribution of the conjugated system. The terminal elimination phase was substantially slower, with kβ values of ~0.03 h^−1^ and terminal half-lives of ~21–22 h, suggesting prolonged systemic persistence. These values for terminal half-life for nanoparticles are consistent with previously reported data for non-conjugated iron oxide nanoparticles of simar size and charge [37]. Exposure, as reflected by AUC_0–∞_, was higher for the iron component compared with the oligo, consistent with the expected retention of the nanoparticle core. Clearance was relatively low for both components (CL ≈ 3.37 mL/h/kg for oligo and 2.35 mL/h/kg for iron), supporting sustained circulation, while the volume of distribution indicated moderate tissue distribution, with oligo showing a slightly higher Vd than iron. Overall, the close agreement in kinetic parameters between oligo and iron supports the stability of the conjugate in vivo and indicates that the oligo remains associated with the iron oxide nanoparticle throughout systemic circulation and elimination.

### 3.2. In Vitro Studies with Established and Patient-Derived Human GBM Cell Lines

**Figure 2:**
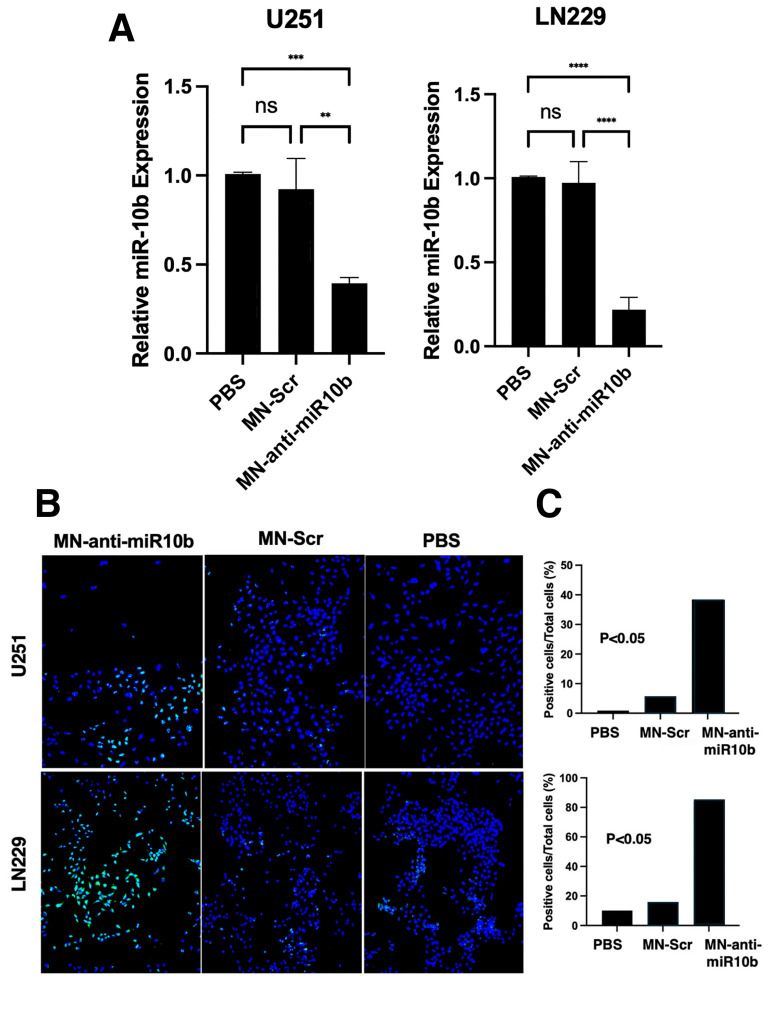
MN-anti-miR10b targeting of miR-10b in GBM cells. (**A**) Cells were incubated with MN-anti-miR10b, MN-Scr or PBS for 48 hrs and subjected to RT-qPCR. Significant inhibition of miR-10b expression was observed in both U251 and LN229 glioblastoma cells (** *p* < 0.01; *** *p* < 0.001; **** *p* < 0.0001; ns—not significant). (**B**) Induction of apoptosis in U251 and LN229 glioblastoma cells upon treatment with MN-anti-miR10b assessed by TUNEL assay. Green—apoptotic cells revealed by TUNEL; blue—DAPI nuclear stain. Bar—100 µm. (**C**) Corresponding quantification of results in (**B**) analyzed using ImageJ2 software.

The MN-anti-miR10b therapeutic consisting of iron oxide nanoparticles conjugated to an antisense oligonucleotide targeting miR-10b or scrambled oligo (MN-Scr) was synthesized and characterized. On average, the therapeutic contained 12–14 oligos/MN, 5–7 Cy5.5 molecules/MN, and was approximately 24.7 nm in size after conjugation with a 6.5 mV Zeta potential, as reported earlier [28]. The profound effect of MN-anti-miR10b on U251 and LN229 human glioblastoma cell viability that we reported earlier [28] resulted in significant downregulation of the target (miR-10b) in these cells (Figure 2A). This effect was also observed in murine (GL261) glioblastoma cells (Supplementary Figure S2A). With the goal of advancing our studies toward future clinical translation, we tested patient-derived cell lines ([33,34], listed in Supplementary Table S1) with MN-anti-miR-10b, which yielded similar results (Supplementary Figure S2B–G). MiR-10b expression in these lines appeared to be significantly (70–97%) inhibited after incubation with MN-anti-miR10b compared to the treatment with MN-Scr or PBS. The observed downregulation of miR-10b in these cells resulted in profound induction of apoptosis in conventional cell lines and patient-derived lines (Figure 2B,C and Supplementary Figure S2H). This was in addition to upregulation of the miR-10b pro-apoptotic targets (HOXD10 and BIM) that we observed in our earlier work [28].

### 3.3. MN-Anti-miR10b Accumulates in Orthotopic GBM Tumors In Vivo

**Figure 3:**
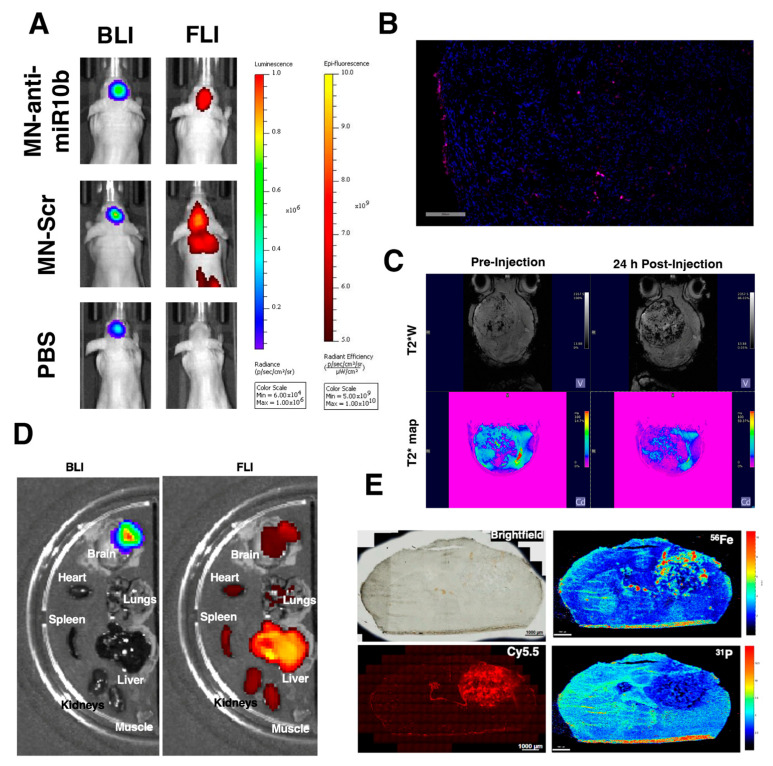
In vivo imaging of therapeutic accumulation in orthotopic GBM tumors. (**A**) In vivo bioluminescence and fluorescence imaging of mice bearing orthotopic U251 tumors and systemically injected with MN-anti-miR10b, MN-Scr, or PBS 24 h after injection. Note the co-localization of the BLI and FLI signals after injections with MN-anti-miR10b or MN-Scr and the absence of the FLI signal after PBS injection. (**B**) Fluorescence microscopy in the Cy5.5 and DAPI channels shows the presence of MN-anti-miR10b in tumor cells, confirming the delivery of the therapeutic. Red—Cy5.5 signal; Blue—DAPI nuclear stain. Scale bar—100 µm. (**C**) T_2_*W (weighted) images and T_2_* maps before and 24 h post-injection showing accumulation of the therapeutic. (**D**) Ex vivo optical imaging of tumors and organs 24 h after administration of the therapeutic shows its localization in the tumor region and other organs of the reticuloendothelial system (RES). (**E**) LA-ICP-TOF-MS and fluorescence imaging revealed the accumulation of iron nanoparticles in the tumor region. Top left: Bright-field microscopy. Bottom left: Cy5.5 microscopy. Top right: LA-ICP-TOF-MS map of ^56^Fe. Bottom right: LA-ICP-TOF-MS map of ^31^P. Scale bar—1000 µm.

The first step in drug development is to demonstrate in vivo accumulation of the therapeutic agent at the target site. We previously used in vivo MR imaging to show the accumulation of unconjugated iron oxide nanoparticles in brain tumors in mice and rats after intravenous injections [38,39]. To determine if MN-anti-miR10b could potentially be efficacious as GBM treatment, we first needed to confirm its ability to cross the disrupted blood–brain barrier and accumulate in the tumor region. To this end, we performed multimodal in vivo imaging of mice orthotopically implanted with luciferase-expressing U251 cells. In vivo bioluminescence (BLI) and fluorescence imaging (FLI) performed 24 h after intravenous injection of Cy5.5-labeled MN-anti-miR10b demonstrated significant co-localization of the BLI signal with the signal from Cy5.5 fluorescence in the brain tumor region (Figure 3A), which was confirmed by fluorescence microscopy in the Cy5.5 channel (Figure 3B). Importantly, the control MN-Scr construct also showed accumulation in the tumor, as expected, whereas mice injected with PBS were devoid of the FL signal. Similar results were obtained with PDX animal models injected with MN-anti-miR10b, in which the FL signal followed the localization of the BLI signal, which was also confirmed by fluorescence microscopy (Supplementary Figure S3A and Supplementary Figure S3B, respectively). Accumulation of TTX-MC138 devoid of Cy5.5 dye in U251 tumors was confirmed using Prussian blue staining (Supplementary Figure S3C).

In addition to the optical imaging capabilities conferred by the Cy5.5 moiety, the superparamagnetic iron oxide core of this therapeutic also serves as a potent T2 magnetic resonance (MR) imaging reporter, enabling MR imaging for drug delivery. Figure 3C shows pre-and postinjection T2*-weighted images and T2* maps of the brain pointing to distinctive areas of signal loss on T2 images associated with the tumors and confirming the accumulation of MN-anti-miR10b 24 h after intravenous injection.

Ex vivo imaging of MN-anti-miR10b biodistribution confirmed the co-localization of bioluminescence and fluorescence signals in the brain (Figure 3D). The biodistribution of iron oxide nanoparticles to other organs, as seen by fluorescence imaging, followed an expected pattern where they accumulated in the reticuloendothelial system (RES) organs for clearance.

To corroborate our in vivo and ex vivo imaging data, we performed laser ablation inductively coupled plasma time-of-flight mass spectrometry (LA-ICP-TOF-MS) (Figure 3E). The tumor region visible in the bright light microphotograph (Figure 3E, top left) showed high levels of iron (Fe^56^) accumulation in comparison to the contralateral normal brain tissue detected by LA-ICP-TOF-MS (Figure 3E, top right). The iron accumulation pattern correlated well with the fluorescence microscopy of the same whole-mounted tissue (Figure 3E, bottom left). Importantly, the phosphorus channel (P31), which was used as a proxy marker for cellular structures in the tissue by detecting phosphorus-containing molecules, such as nucleic acids and lipids, did not show any specific signal with LA-ICP (Figure 3E bottom right).

### 3.4. MN-Anti-miR10b Improves Survival in Orthotopic Models of Glioblastoma

**Figure 4:**
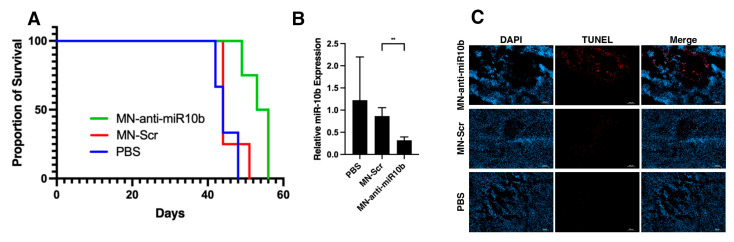
Therapeutic studies with MN-anti-miR10b in an orthotopic U251tumor model. The treatment groups included intravenous injections of MN-anti-miR10b (10 mg oligo/kg, n = 6), MN-Scr (10 mg oligo/kg, n = 5), or PBS (n = 6). Treatment was continued for 8–10 weeks or until the animals became moribund. (**A**) Kaplan–Meier survival analysis showed improved survival in the group that received MN-anti-miR10b (54.4 days, *p* < 0.05) compared to groups that received MN-Scr (44 days) or PBS (44 days). (**B**) qRT-PCR analysis of tumor-bearing brains showing significant downregulation of miR-10b in animals injected with MN-anti-miR10b (** *p* < 0.01). (**C**) TUNEL staining of frozen tumor sections showing induced apoptosis in tumor cells of animals injected with MN-anti-miR10b in vivo. Blue—DAPI nuclear stain; red—TUNEL. Scale bar—100 µm.

**Figure 5:**
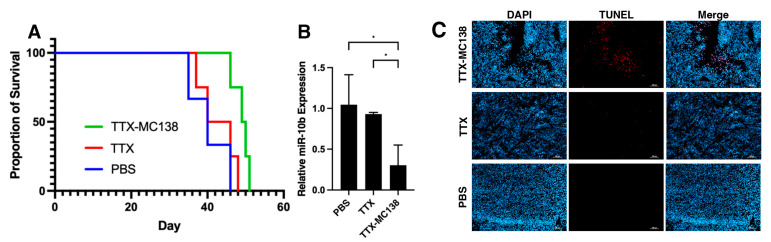
Therapeutic studies with TTX-MC138 in orthotopic U251 tumor model. The treatment groups included intravenous injections of TTX-MC138 (10 mg oligo/kg, n = 6), TTX (20 mg Fe/kg, n = 7), and PBS (n = 7). Treatment was continued for 8–10 weeks or until the animals became moribund. (**A**) Kaplan–Meier survival analysis showed improved survival in the group that received TTX-MC138 (49.5 days, *p* < 0.05) compared to groups that received TTX (43 days) or PBS (40 days). (**B**) TTX-MC138 engages with the target and inhibits the expression of miR-10b in tumor-bearing animals (* *p* < 0.05). (**C**) TUNEL staining of frozen tumor sections showing significant induction of apoptosis in tumor cells of animals injected with TTX-MC138 in vivo (*p* < 0.05). Blue—DAPI nuclear stain; red—TUNEL. Scale bar—100 µm.

Having established that MN-anti-miR10b accumulates in GBM tumors in vivo, we performed therapeutic studies to determine whether these effects indeed confer survival benefits in mouse models. Mice bearing orthotopic U251 tumors received intravenous injections of MN-anti-miR10b or control (PBS and MN-Scr) beginning on day 7 post implantation. Similarly, three other groups of animals received TTX-MC138, MN, or PBS intravenously. At the end of the study, the animals were sacrificed, and the tumors were processed for qRT-PCR and histology. Kaplan–Meier survival analysis showed that survival in the group that received MN-anti-miR10b was significantly longer (54.4 days, *p* < 0.01) than in groups that received MN-Scr (44 days) or PBS (44 days) (Figure 4A). Analysis of miR-10b expression in tumor-bearing brains revealed that treatment with MN-anti-miR-10b significantly reduced miR-10b levels compared to the MN-Scr (>63%, *p* = 0.001) or PBS (>73%, *p* = 0.003) groups (Figure 4B). There was no statistically significant difference in miR-10b expression between MN-Scr- and PBS-treated mice, indicating that the observed effect was miR-10b-specific. Tumor cell death was 3.5 times more pronounced in animals treated with MN-anti-miR10b than in those treated with PBS (*p* = 0.01) (Figure 4C). In the group injected with TTX-MC138, the difference in median survival between TTX-MC138, TTX, and PBS was similar (49.5, 43, and 40 days, respectively, with *p* = 0.03 for TTX-MC138 vs. TTX and *p* = 0.03 for TTX-MC138 vs. PBS, Figure 5A). There was a corresponding decrease in miR-10b expression in the TTX-MC138-treated group compared to the TTX (>67%, *p* = 0.04) or PBS (>71%, *p* = 0.04) control groups (Figure 5A,B). Analysis of tumor sections showed that in mice treated with TTX-MC138 apoptosis increased approximately five times compared with PBS-treated mice (*p* < 0.0001), as indicated by TUNEL staining (Figure 5C). Interestingly, BLI demonstrated steady increase in bioluminescence throughout the course of the study with both formulations (Supplementary Figure S4A,B) indicating that factors such as tumor necrosis, hypoxia, variable luciferin delivery across the blood–brain barrier, and treatment-associated inflammation and edema causing “pseudo-progression” can all significantly influence BLI signal independently of true tumor progression or regression. As expected, animal body weight continued to decline throughout the study (Supplementary Figure S4C,D) indicating the disease progression despite the observed difference in survival. Importantly, histopathological analysis revealed no treatment-related morphological changes in major organs (Supplementary Figure S5), supporting the safety of the therapeutic and consistent with our previous findings [29,31,32].

## 4. Discussion

Since the initial implication of miRNAs in tumorigenesis, modulation of miRNA expression has been explored as a therapeutic approach for various cancer types [40]. In a landmark study by Ma et al., the role of miR-10b in promoting tumor invasion and migration was first elucidated in triple-negative breast cancer [41]. Overexpression of miR-10b in GBM samples was first reported in 2005 in a study evaluating the expression of different miRNAs in this cancer type [42]. Since then, miR-10b has been extensively studied in GBM and found to be critical for invasion and migration, and most importantly, GBM cell survival [5,14,28,43]. Although numerous studies have been carried out to inhibit miR-10b in GBM, none have reached the point of clinical translation.

In the present study, for the treatment of orthotopic GBM tumors, we employed MN-anti-miR10b, a superparamagnetic iron oxide nanoparticle carrying 23-mer anti-miR-10b LNAs oligo, as well as TTX-MC138, an oligo-nanoparticle conjugated miR-10b inhibitor that is used in clinical trials for the treatment of patients with advanced solid (non-GBM) cancers. Since drug delivery to GBM presents a significant clinical challenge, it is imperative to demonstrate the tumoral accumulation of the therapeutic agent. We reasoned that with a hydrodynamic size of approximately 20 nm and a long circulation half-life, the nanoparticle platform is well suited for accumulation in GBM tumors, which often exhibit compromised vasculature and enhanced permeability and retention (EPR) effects. The EPR effect has been previously exploited by us and others for imaging brain tumors using nanomaterials (“passive” targeting) in preclinical and clinical settings [38,39,44,45,46]. It is noteworthy that MN-anti-miR10b accumulates in established brain metastases from breast cancer, suggesting that sufficient penetration can be achieved in lesions in which the blood–brain barrier is disrupted [32], which we expected to show in our studies. Indeed, our data demonstrated that MN-anti-miR10b was successfully delivered to the tumor region, likely due to its ability to cross the disrupted blood–brain barrier. This observation was confirmed using three independent modalities (FLI, MRI, and mass spectrometry), thereby de-risking the clinical translation of our studies. Other organs that accumulated the therapeutic included the liver, spleen, and kidneys, which is in line with our previously reported biodistribution data [47] and patterns for analogous nanoparticles used for imaging [48,49,50,51,52]. Importantly, our stability and pharmacokinetic studies showed that the oligo-nanoparticle conjugate remains stable in plasma, with long circulation time ensuring appreciable tumoral accumulation. The nanoparticles that serve as delivery vehicles are eventually taken up by cells of the reticuloendothelial system within these organs, where they are subsequently broken down for clearance [53,54]. The iron from the core of the nanoparticles is stored in secondary low pH lysosomes within the cytoplasm, ultimately degraded and enters the endogenous iron pool, whereas dextran from the nanoparticle coating is cleared through the kidneys [48,55]. It is important to note that the design of the nanoparticle–ASO conjugate ensures efficient delivery of the oligonucleotide to target cells. Once internalized, the disulfide bond dissociates in the acidic intracellular environment, releasing the oligonucleotides into the cytoplasm, where they engage in RNA interference (RNAi). We have previously confirmed this mechanism using fluorescently labeled ASOs [30,56]. The profound downregulation of miR-10b observed across various cancer models provided further evidence of the successful in vivo delivery of the ASO by the nanoparticles to tumors in small and large animals [29,30,31,32,35]. We also need to mention that as with the vast majority of cancer therapies where multiple doses are required, we expect that TTX-MC138 will be administered through multiple cycles given that only a portion of cells is targeted after each injection in this hard-to-treat cancer (Figure 2B and Supplementary Figure S2B). Importantly, accumulation of MN-anti-miR10b and any potential miR-10b downregulation in non-tumor sites are not expected to have deleterious effects, as studies showed that miR-10b deficient mice develop normally [57]. Notably, the ability to detect nanoparticle accumulation using a clinically relevant imaging modality (MRI) further demonstrates its potential as a drug delivery platform for GBM.

In addition to performing studies with established cell lines, we performed pilot studies in patient-derived models, which represent a critical component of drug development because of their high translational relevance and ability to closely mirror the complexity of human GBM. Unlike conventional cell line-based models, PDXs maintain the genetic, epigenetic, and histopathological features of the original tumors, including intratumoral heterogeneity and treatment resistance patterns. Although therapeutic studies in these models are still pending, we took the first step in establishing their feasibility by demonstrating significant miR-10b downregulation across all lines. Importantly, downregulation of miR-10b in these lines was independent of the baseline miR-10b expression levels, MGMT promoter status or response to TMZ or radiation therapy (Supplementary Table S1). Indeed, in our previous study we showed that expression of MGMT, the main regulator of TMZ resistance in glioblastomas remained unchanged after treatment [28]. This observation likely reflects the role of miR-10b as an upstream regulatory miRNA that controls core oncogenic programs rather than therapy-specific resistance pathways. MGMT promoter status primarily influences DNA repair capacity [58] and does not regulate miRNA expression or antisense-mediated inhibition. In addition, sequence-specific anti-miR oligonucleotides achieve target suppression largely independent of endogenous miRNA abundance. Finally, cellular responses to TMZ or radiation reflect downstream stress and repair mechanisms, whereas miR-10b inhibition occurs upstream of these processes. Together, these factors explain the consistent miR-10b downregulation observed across molecularly and therapeutically diverse GBM cell lines. This is in line with our previous studies in metastatic breast cancer where MN-anti-miR10b downregulated miR-10b in various breast cancer types independent of the tumor receptor status [59]. Notably, we also achieved significant in vivo accumulation of the therapeutic agent within these tumors, further supporting the rationale for proceeding with therapeutic studies.

Our therapeutic studies demonstrated that both MN-anti-miR10b and TTX-MC138 formulations improved survival in orthotopic GBM tumors, with appreciable inhibition of the target and induction of tumor cell apoptosis. However, we need to address the limitations of the current study demonstrating relatively modest survival benefit despite the strong miR-10b inhibition. This may reflect the highly aggressive and adaptive nature of GBM. miR-10b regulates key oncogenic pathways related to invasion and migration, and its suppression by monotherapy is expected to delay progression rather than achieve complete tumor eradication. In addition, GBM exhibits substantial pathway redundancy and intratumoral heterogeneity, allowing residual tumor cells to adapt despite effective target engagement. Heterogeneous intratumoral delivery resulting from a dynamic tumor microenvironment that is continuously reprogrammed by tumor cells [60], together with the stringency of survival as an endpoint in orthotopic GBM models, further contributes to the limited survival gains observed. These findings suggest that miR-10b inhibition may be most effective as part of a combination strategy rather than a monotherapy that we plan to conduct in the future along with investigation of the appropriate downstream targets known for miR-10b.

Since IND-enabling studies as well as pharmacokinetics, biodistribution, and required toxicity studies for TTX-MC138 have already been completed, we are well positioned to incorporate it into our future studies, including those in PDX models, where we plan to combine it with standard-of-care treatments such as temozolomide and radiation therapy. The clinical readiness of TTX-MC138, along with the success demonstrated in this preclinical study, significantly facilitates its transition into clinical trials and potential therapeutic applications in GBM patients.

## Figures and Tables

**Table 1:** Pharmacokinetic parameters of the therapeutic. Rats (n = 3/time point, m = 10 time points) were intravenously injected with TTX-MC138 (30 mg oligo/kg). Plasma was analyzed for the presence of oligo and iron.

Parameter	Oligo	Iron
kα (h^−1^)	0.145 ± 0.004	0.144 ± 0.005
t_1/2_,α (h)	4.8 ± 0.13	4.8 ± 0.16
kβ (h^−1^)	0.033 ± 0.001	0.031 ± 0.001
t_1/2_,β (h)	21.0 ± 0.64	22.2 ± 0.72
A (ng/mL)	328,041 ± 14,500	449,462 ± 19,000
B (ng/mL)	218,694 ± 11,000	299,641 ± 14,500
AUC_0–∞_ (ng·h/mL)	8,889,443 ± 275,000	12,789,145 ± 350,000
CL (mL/h/kg)	3.37 ± 0.10	2.35 ± 0.06
Vd (mL/kg)	102 ± 3.2	75.8 ± 2.5

## Data Availability

The original contributions presented in the study are included in the article, further inquiries can be directed to the corresponding author.

## Supplementary Materials

The following supporting information can be downloaded at: https://www.mdpi.com/article/10.3390/jfb17010015/s1, Supplementary Table S1. Patient-derived xenograft cell lines used in the study. Supplementary Figure S1. Bioluminescence images of animals enrolled in study 1 (MN-anti-miR10b, MN-Scr and PBS groups) and study 2 (TTX-MC138, TTX and PBS), Day 7 after tumor implantation, Day 0 of treatment. Supplementary Figure S2. (A) Inhibition of miR-10b in murine glioblastoma cell line GL261 after incubation with MN-anti-miR10b. Cells were incubated with MN-anti-miR10b, MN-Scr or PBS for 48 hrs and subjected to RT-qPCR. Significant inhibition of miR-10b expression was observed in all cell lines tested. Incubation with MN-Scr and PBS did not produce any effect on the target. (B–G) Inhibition of miR-10b in patient-derived cell lines (listed in Supplementary Table S1) after incubation with MN-anti-miR10b. Incubation with MN-Scr and PBS did not produce any effect on the target. (H) Induction of apoptosis in patient-derived cell lines after incubation with MN-anti-miR10b (HF2927 shown). ** *p* < 0.01, *** *p* < 0.001, **** *p* < 0.0001. Supplementary Figure S3. (A) Representative in vivo BLI and FLI imaging of MN-anti-miR10b accumulation in PDX orthotopic tumors (HF3016 shown). Animals were injected with MN-anti-miR10b (10 mg oligo/kg) and imaged 24 h later. (B) Fluorescence microscopy in the Cy5.5 and DAPI channels shows the presence of MN-anti-miR10b in tumor cells confirming the delivery of the therapeutic. Scale bar—100µm. (C) Prussian blue staining confirms accumulation of TTX-MC138 in orthotopic U251 tumors. Animals were injected with TTX-MC138 (10 mg oligo/kg) and sacrificed 24 h later. Arrows point to blue deposits staining non-heme iron in tissues. Scale bar—100µm. Supplementary Figure S4. Changes in BLI signal (A,B) and body weight (C,D) in animals injected with MN-anti-miR10b (A,C) and TTX-MC138 (B,D) with appropriate controls. Survival curves are shown in Figs. S4 and S5 respectively. Supplementary Figure S5. Histopathology of major organs at necropsy after treatment with MN-anti-miR10b shows no treatment-related morphological changes supporting the safety of the therapeutic.
