# Supplementary material for: Nanotherapy Targeting miR-10b Improves Survival in Orthotopic Glioblastoma Models
Source: J Funct Biomater. 2025 Dec 26;17(1):15. doi: 10.3390/jfb17010015 (PMC12843090; doi:10.3390/jfb17010015)
Supplement: Supplementary file 1 [file jfb-17-00015-s001.zip › jfb-4026141-supplementary.pdf]

Supplementary Materials

Nanotherapy targeting miR-10b improves survival in orthotopic glioblastoma models

Bryan D. Kim[1,3], Ming Chen[1,2], Sujan K. Mondal[1,2], Elizabeth Kenyon[1,2], Christiane L. Mallett[2,4], Ana deCarvalho[5], Zdravka Medarova[6] and Anna Moore[1,2*]

[1]Precision Health Program, Michigan State University, East Lansing, MI, United States. [2]Department of Radiology, College of Human Medicine, Michigan State University, East Lansing, MI, United States.

[3]Department of Biomedical Engineering, College of Engineering, Michigan State University, East Lansing, MI, United States.

[4]Institute for Quantitative Health Science and Engineering, Michigan State University, East Lansing, MI, United States.

[5]Henry Ford Health, Detroit, MI, United States.

[6]Transcode Therapeutics Inc., Woburn, MA, United States.

Supplementary Table S1. Patient-derived xenograft cell lines used in the study.

	Clinical and demographic data							Orthotopic PDX median survival (days)	Response to therapy and miR-10b expression		
**Surgical specimen ID**	**Path**	**Brain Location**	**Gender**	**A.D.**	**TTP1 (days)**	**OS (days)**	**MGMT promoter**		**TMZ**	**RT**	**miR-10b expression**
HF3016	GBM - ND	R Temporal	M	45	88	646	U	43	Resistant	Medium	high
HF3253	GBM - ND	L Frontal	F	82		68	U	29	Sensitive	Sensitive	high
HF3077	GBM - ND	L Parietal	F	56	54	465	U	134	Sensitive	Resistant	high
HF2354	GBM - ND	L Frontal	M	61	60	196	U	87	Resistant	Sensitive	medium
HF3035	GBM - ND	R Parietal	F	54	196	352	U	127	Sensitive	Resistant	medium
HF2927	GBM - ND	L Frontal	F	55	11	664	M	52	Sensitive	Sensitive	low

ND, newly diagnosed; A.D., age at diagnosis; TTP1, time to first progression; OS, overall survival; U, unmethylated; M, methylated.

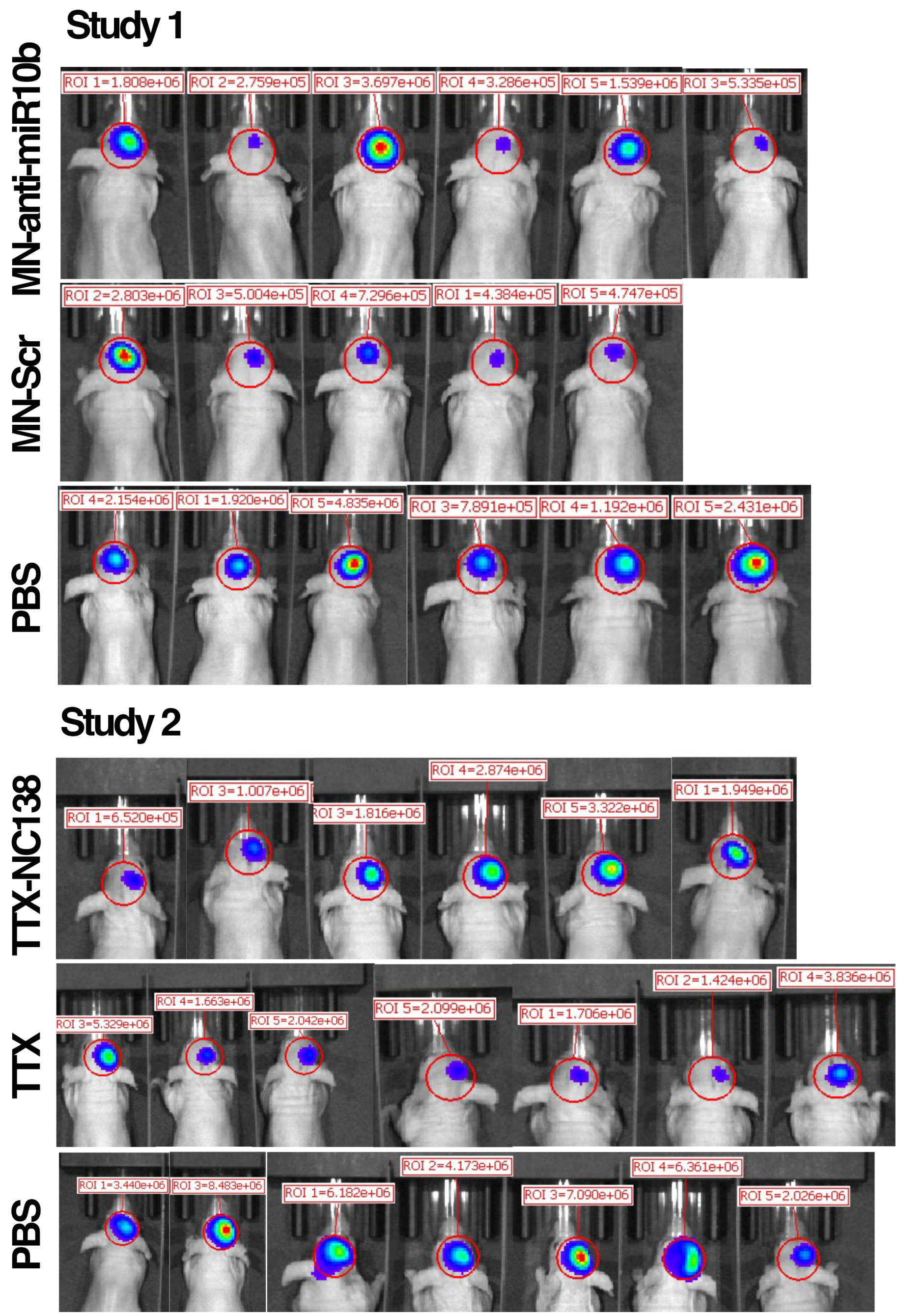

Supplementary Fig. S1. Bioluminescence images of animals enrolled in study 1 (MN-anti-miR10b, MN-Scr and PBS groups) and study 2 (TTX-MC138, TTX and PBS), Day 7 after tumor implantation, Day 0 of treatment.

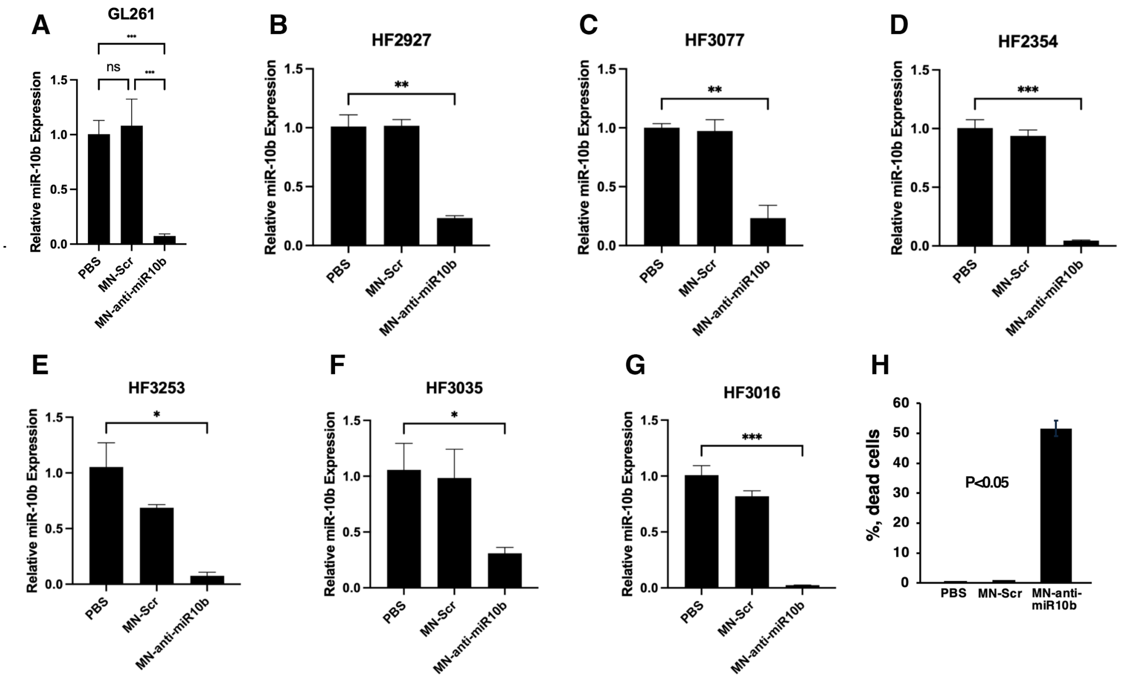

Supplementary Fig. S2. (**A**) Inhibition of miR-10b in murine glioblastoma cell line GL261 after incubation with MN-anti-miR10b. Cells were incubated with MN-anti-miR10b, MN-Scr or PBS for 48 hrs and subjected to RT-qPCR. Significant inhibition of miR-10b expression was observed in all cell lines tested. Incubation with MN-Scr and PBS did not produce any effect on the target. (**B-G**) Inhibition of miR-10b in patient-derived cell lines (listed in **Supplementary Table S1**) after incubation with MN-anti-miR10b. Incubation with MN-Scr and PBS did not produce any effect on the target. (**H**) Induction of apoptosis in patient-derived cell lines after incubation with MN-anti-miR10b (HF2927 shown). ** p<0.01, *** p<0.001, **** p<0.0001.

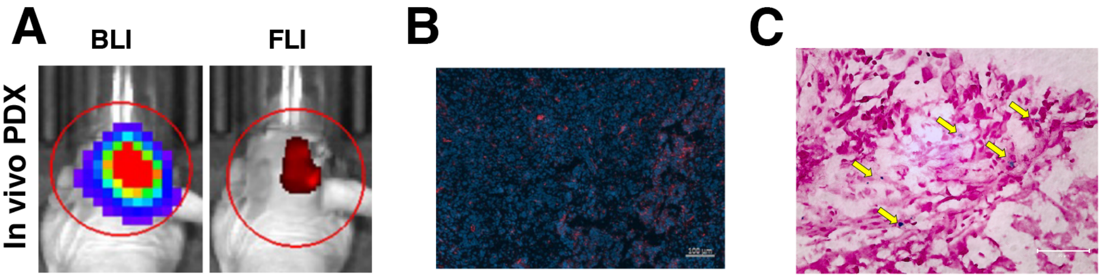

Supplementary Figure S3. (**A**) Representative *in vivo* BLI and FLI imaging of MN-anti-miR10b accumulation in PDX orthotopic tumors **(**HF3016 shown). Animals were injected with MN-anti-miR10b (10 mg oligo/kg) and imaged 24 h later. (**B**) Fluorescence microscopy in the Cy5.5 and DAPI channels shows the presence of MN-anti-miR10b in tumor cells confirming the delivery of the therapeutic. Scale bar – 100µm. (**C**) Prussian blue staining confirms accumulation of TTX-MC138 in orthotopic U251 tumors. Animals were injected with TTX-MC138 (10 mg oligo/kg) and sacrificed 24 h later. Arrows point to blue deposits staining non-heme iron in tissues. Scale bar – 100µm.

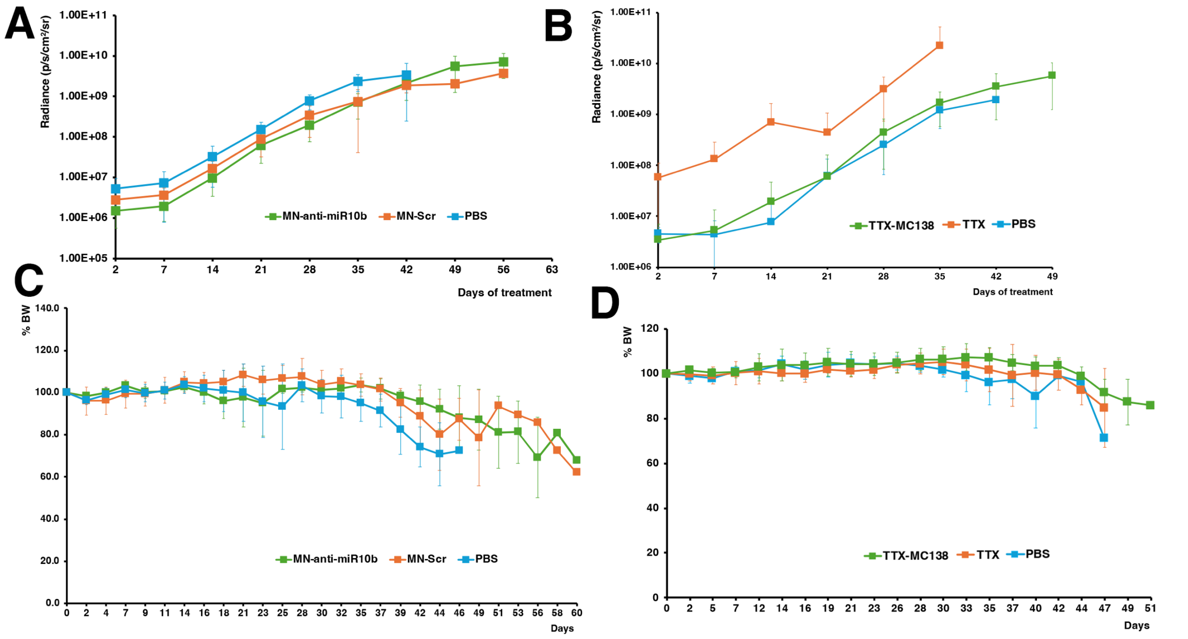

Supplementary Figure S4. Changes in BLI signal **(A, B)** and body weight **(C, D)** in animals injected with MN-anti-miR10b **(A, C)** and TTX-MC138 **(B, D)** with appropriate controls. Survival curves are shown in Figs. 4 and 5 respectively.

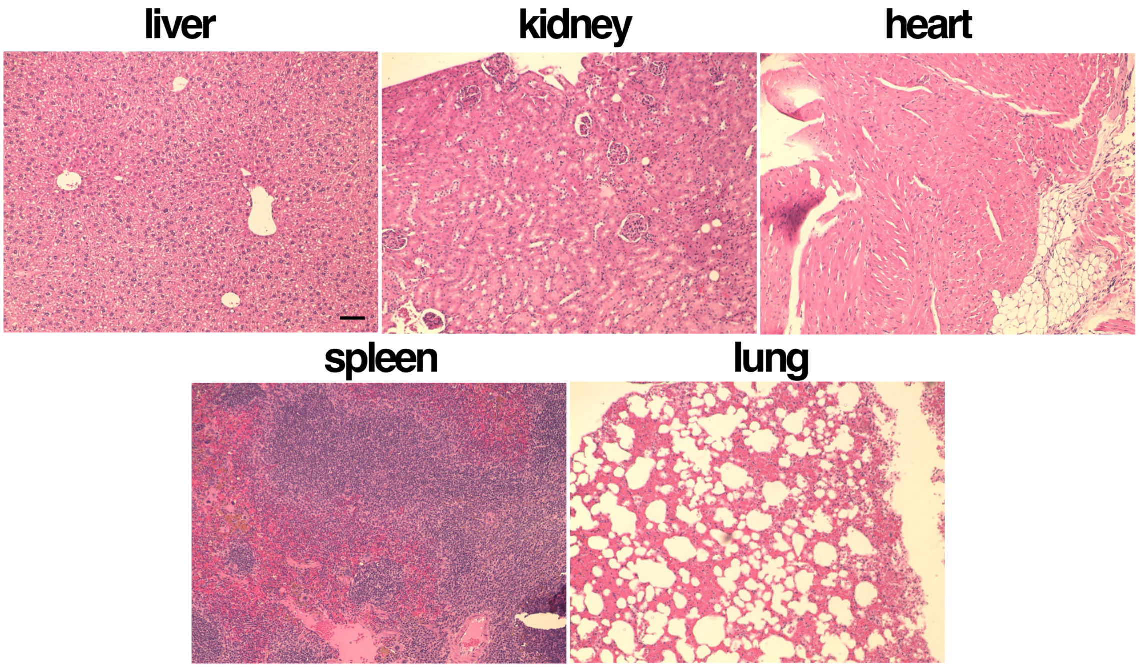

Supplementary Figure S5. Histopathology of major organs at necropsy after treatment with MN-anti-miR10b shows no treatment-related morphological changes supporting the safety of the therapeutic.
